# Observation of targeted gold nanoparticles in nasopharyngeal tumour nude mice model through dual‐energy computed tomography

**DOI:** 10.1049/nbt2.12035

**Published:** 2021-03-11

**Authors:** Sara Khademi, Ali Shakeri‐Zadeh, Razieh Solgi, Hosein Azimian, Hossein Ghadiri

**Affiliations:** ^1^ Department of Radiology Technology School of Paramedical Sciences Mashhad University of Medical Sciences Mashhad Iran; ^2^ Medical Physics Department School of Medicine Iran University of Medical Sciences (IUMS) Tehran Iran; ^3^ Department of Medical Physics and Biomedical Engineering Tehran University of Medical Sciences Tehran Iran; ^4^ Medical Physics Research Center Mashhad University of Medical Sciences Mashhad Iran

## Abstract

This study was performed to specify the efficiency of imaging nanoparticle concentration as contrast media in dual‐energy computed tomography (DECT). Gold nanoparticles (AuNPs) and gold nanoparticles‐conjugated folic acid through cysteamine (FA‐Cya‐AuNPs) were both considered as contrast agents. Characterization of NPs was performed using Dynamic Light Scattering (DLS) and zeta potential. The hemocompatibility of NPs was confirmed by different blood parameters such as white blood cell, red cell distribution width, hemoglobin, lymphocytes counts and haemolysis assay. DECT algorithm was confirmed using calibration phantom at different concentrations of NPs and tube potentials (80 and 140 kVp). Then, DECT was used to quantify the concentration of both AuNPs and FA‐Cys‐AuNPs in human nasopharyngeal cancer cells. Mice were injected with non‐targeted AuNPs and targeted AuNps at a concentration of 3 × 10^3^ μg/ml. Then, they were scanned with different tube potentials. The concentration of nanoparticles in the various organs of nude mice was measured through DECT imaging and inductively coupled plasma mass spectrometry (ICP‐MS) analysis. The results of DECT images were compared with ICP‐MS analysis and indicated that they were approximately similar. In sum, FA‐Cys‐AuNPs can be a proper candidate for targeted contrast media in DECT molecular scanning of human nasopharyngeal tumours.

## 1 INTRODUCTION

Dual‐energy computed tomography (DECT) imaging is an efficient technique that permits for optional quantification and visualization of materials with high atomic in two scans [1]. In DECT, two various X‐ray beam energies were used to obtaininformation of two images of the animals, to permit the assessment of energy‐related alterations in the X‐ray intensity attenuation of different materials [2]. Nowadays, trends in X‐ray scanning are relying on material decomposition, identification and separation in images [3]. DECT applies the technique of subtraction, or to be more particular an algorithm that contrasts pixel Hounsfield unit (HU) value between image data, to identify materials relied on their X‐ray energy‐related intensity attenuation [4]. Therefore, DECT can analyse both diagnostic images as routine and material based on density images. Since materials based on k‐edges have specific X‐ray attenuation intensity (XAI) profiles at various potential energy levels pursuant to their linear attenuation coefficient (LAC), DECT can apply mathematical different algorithms to evaluate tissues when exposed to low and high X‐ray energy polychromatic beam of the potential tube [5]. Materials or tissues with low atomic numbers (Z) (such as water) reveal small variations in XAI between low and high X‐ray energy beam in contrast with materials with high atomic number (such as iodine) [6]. Since the increasing atomic number and decreasing density of materials in X‐ray attenuation reveal the same effect of decreasing the atomic number and increasing density, therefore the sensitivity of CT with single energy is low [7]. Since the tumours commonly have a similar density (*ρ*) as their nearly tissues, contrast media are essential for tumour cells to distinguish and analyse using DECT. Contrast media in X‐ray imaging modality prepare both anatomical (such as vascular) and functional (such as perfusion) data. Iodinated contrast media are the most generally applied contrast materials. Lately, gold nanoparticle (AuNPs) contrast agents have been reported for imaging. Au has an atomic number of *Z* = 79 and electron density of *ρ* = 19.32 g/cm^3^ giving it a higher XAI than iodine contrast agent (*Z* = 53 and *ρ* = 4.9 g/cm^3^) [8]. Biocompatibility is another advantage of Au as a contrast media for in vivo applications [9]. AuNPs were first explained by Hainfield et al. [10]. Other studies have also shown a preference for Au‐based nanoprobes in contrast to iodinated media [4, 11, 12]. Boote et al. [13] have revealed the usage of AuNPs as a contrast media with a hospital CT. They described that concentrations of Au in tissues elevated the CT values in the liver by nearly 22 and 27 HU at 80 and 140 kVp, respectively [5]. This information was consistent with HU variations revealed for the same concentrations in the physical phantom. Jackson et al. [12] studied the advantages of AuNPs in image contrast enhancement relative to conventional iodinated contrast media. Their results demonstrated an enhancement of 89% in CNR at low X‐ray energies. On the other hand, no significant variation in the improvement of CNR was revealed at X‐ray tube potentials that are usually applied for angiography (nearly 80 kVp) which may be because of the effect of the k‐edge for a conventional iodine contrast agent. As imaging nanoprobes, NPs associated with different targeting can simplify molecular imaging (MI) with DECT. Folic acid (FA) or folate is one of the important and maximum targeting receptors on nasopharyngeal cancer cells. Therefore, for evaluating DECT targeting imaging, in the previous study, AuNPs were conjugated with FA through cysteamine (FA‐Cys‐AuNPs)[14, 15]. In this study, we have sought to apply an improved methodology for recognizing AuNPs and FA‐Cys‐AuNPs concentration in the body of animals using DECT. It can be applied to prevent dissection of organs instead of ICP analysis.

## 2 MATERIALS AND METHODS

### 2.1 Materials

The nanoparticles were synthesized as reported by the previous work. Fetal bovine serum was purchased from BioSera (United Kingdom). RPMI was obtained from GIBCO (Germany) and ketamine, xylazine, ethylenediaminetetraacetic acid (EDTA), penicillin–streptomycin, dimethyl sulfoxide (DMSO), trypsin, cysteamine, gold salts and folic acid were prepared from Sigma‐Aldrich Corp. (St. Louis, MO, USA).

### 2.2 Characterization techniques

Dynamic Light Scattering (DLS) was done using a Zeta sizer Nano ZSP to investigate the hydrodynamic size of NPs. Zeta potential was performed to measure the surface charge of NPs. The concentrations of NPs were evaluated by inductively coupled plasma mass spectrometry (ICP‐MS).

### 2.3 CT scans of calibration phantom

In the previous study, for the calibration of phantom, the X‐ray attenuation of the five cavities that were filled by vials containing different concentrations of FA‐Cys‐AuNPs (250**,** 500, 1000, 1500 and 2000 μg/ml) was measured at two different energy levels (80 and 140 kVp) [2]. Then, a decomposition method as a post‐reconstruction was performed to evaluate the concentration. Values for the coefficients of the sensitivity at every energy (*CTW, 80*, *CTW, 140*, *CTAu, 80* and *CTAu, 140*) were determined using a calibration phantom made of PMMA (poly (methyl methacrylate)) as explained previously. As represented in Formula (1), there are two uncertain for each voxel in this process, one of them for concentration of water (*C*_W_), and another one for FA‐Cys‐AuNPs concentration *C*_Au_, that is calculated as mg/ml. The two measurements of CT value (*E*1) and (*E*2) demonstrate the level of absorption of two tube potentials.

(1) $$\left[\begin{array}{l} C_{w}\\ C_{\text{Au}} \end{array}\right]={\left[\begin{array}{ll} e_{w\;E1} & e_{\text{Au}\;E1}\\ e_{w\;E2} & e_{\text{Au}\;E2} \end{array}\right]}^{-1}\left[\begin{array}{l} {\text{CT}}_{E1}\\ {\text{CT}}_{E2} \end{array}\right]$$

**FIGURE 1:**
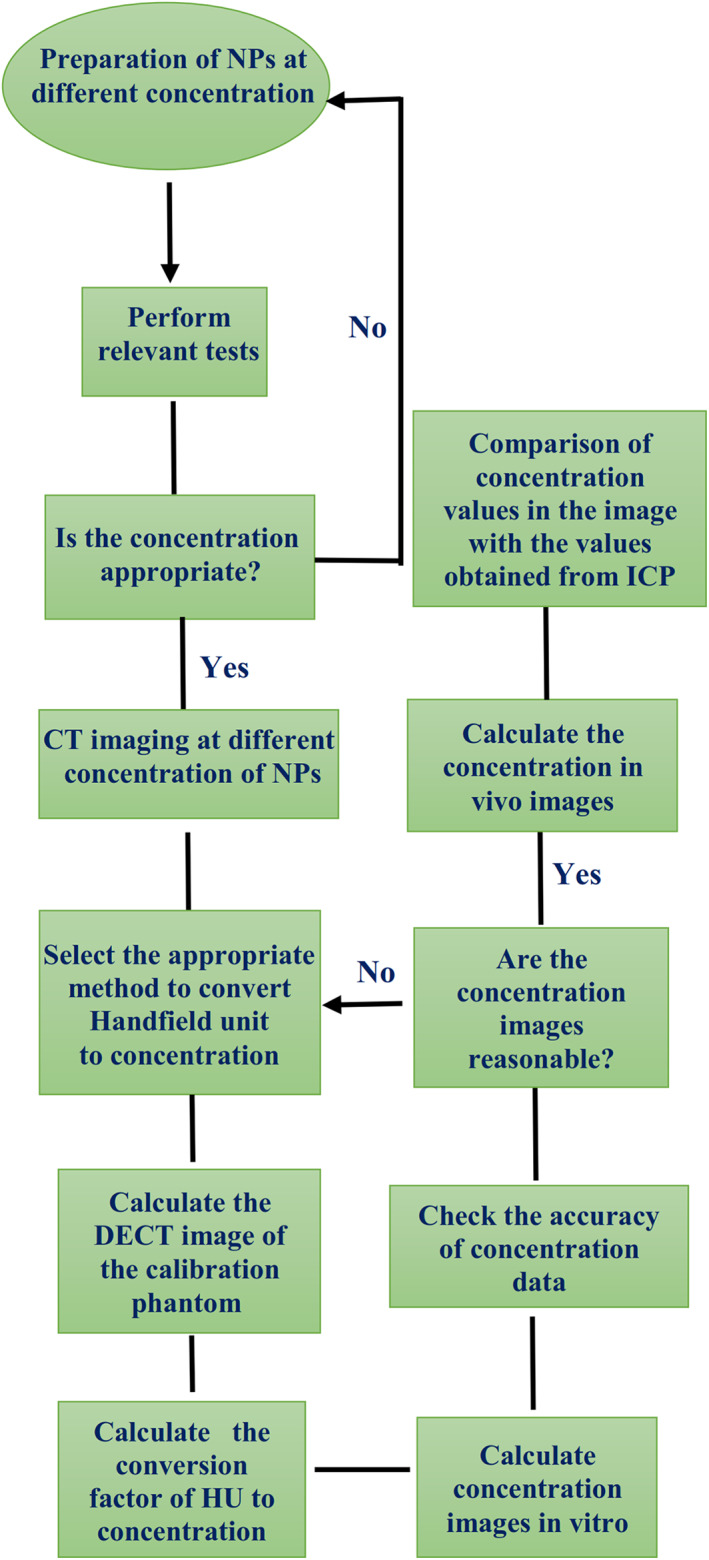
Pathway of DECT to access the images that based concentration of NPs

After that, the above equation was evaluated and put in MATLAB software. The changes in the concentration of calibration phantom were confirmed by ICP‐MS analysis. In this part, a linear correlation (LC) with the slope of the line demonstrated the coefficient of sensitivity (HU/mg ml^**−**1^). It was the correlation between HU and the concentration. Figure 1 represents the pathway of DECT to access the images‐based concentration of NPs.

### 2.4 Haemocompatibility of the FA‐Cys‐AuNPs

The toxicity of FA‐Cys‐AuNPs was examined by haematology parameters. NPs with a concentration of 3 × 10^3^ were injected in nude mice through intravenous injection (IV). After 14 days, the blood was collected in tubes covered by EDTA. The parameters were red blood cell (RBC) count, white blood cell count, lymphocytes (LYM) and haemoglobin (HGB)[16].

Another test to evaluate the toxicity of NPs was haemolysis (rupture of RBCs). The method has relied on the release of haemoglobin that can be examined by the spectrophotometer. Fresh human blood cells were acquired and stabilized with EDTA. First, 2 mL of whole blood was added into 10 mL of PBS (pH ¼ 7.4), centrifuged at 1000 rpm for 15 min to separate human red blood cells (HRBCs) from plasma. The HRBCs were washed with PBS three times and then resuspended in 15 mL of PBS. After that, 200 µL of diluted RBCs suspension was mixed with FA‐Cys‐AuNPs at various concentrations of Au (200, 300, 400, 500 and 600 μM) using gentle shaking and incubated at room temperature (37^º^C) for 4 h. Then, the suspensions were centrifuged to collect serum for 5 min. The serum was again centrifuged to remove NPs (9000 rpm for 10 min). Afterwards, 100 µL of serum of samples was transferred to a plate. UV–Vis spectrophotometer at 540 nm was used to calculate free haemoglobin (FH) in the serum [17]. The amount of haemolysis was obtained using Equation (2):

(2) $$\text{Hemolysis}=\frac{\left(\text{abs}\;\left(\text{sample}\right)-\text{abs}\;\left(\text{negative}\;\text{control}\right)\right)}{\left(\text{abs}\;\left(\text{postive}\;\text{control}\right)-\text{abs}\;\left(\text{negative}\;\text{control}\right)\right)}\;$$

Standard deviations (SD) were measured from the triplicate samples.

### 2.5 In vivo DECT imaging of a nasopharyngeal tumour model

**FIGURE 2:**
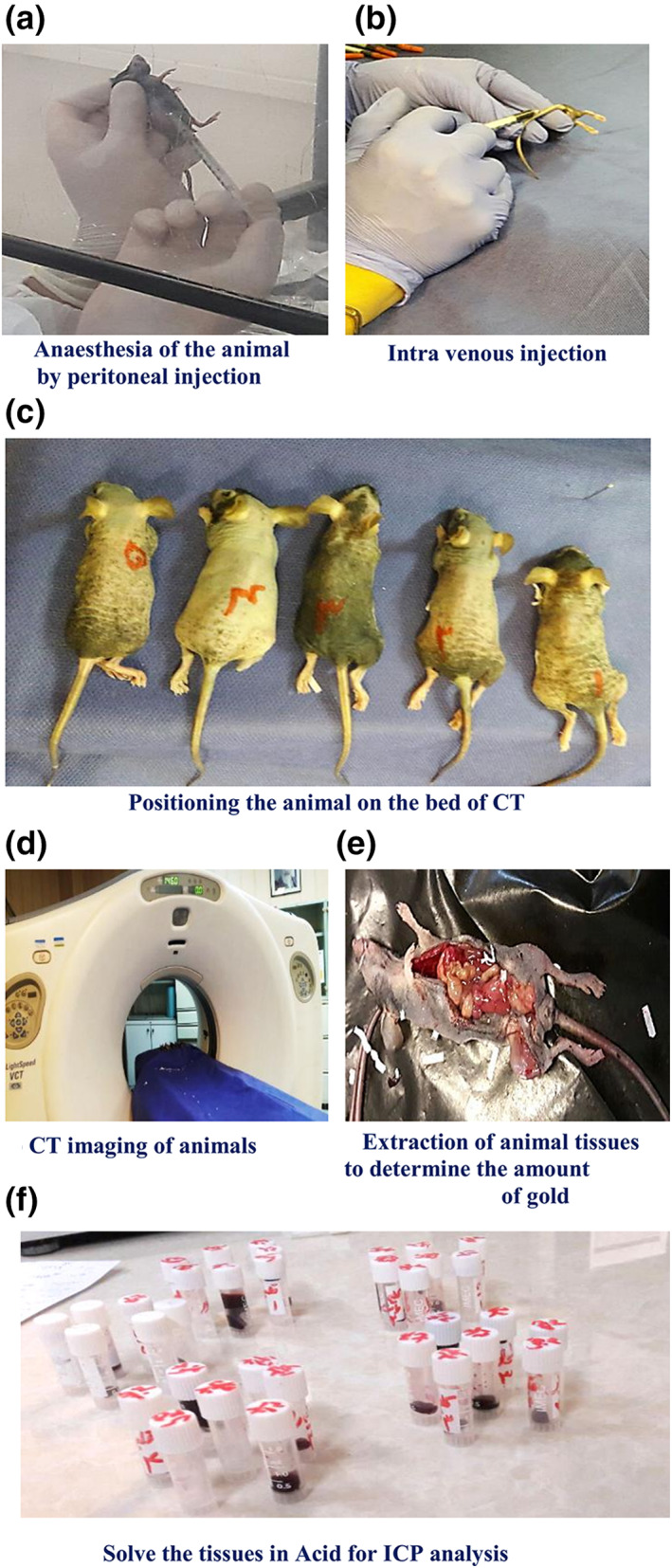
Pathway of preparation and quantification of in vivo imaging

Animal handling and care were performed according to protocols approved by the ethics of the animal research committee of Tehran University of Medical Sciences (TUMS). Four weeks male nude mice (20–22 g) were subcutaneously injected with approximately 10 × 10^6^ cells per mice onto the right flank. After 3 weeks, when the mean KB tumour volume was 0.1 cm^3^, we started the in vivo imaging. The tumour‐bearing nude mice were scanned by a 64‐slice CT modality at two energy levels (80 and 140 kVp) with parameters (pitch = 1, mAs = 250 and slice thickness = 0.625 mm). CT imaging was done before and after IV injections of FA‐Cys‐AuNPs and AuNPs at a concentration of 3 × 10^3^ μg/ml in the tail vein. Three hours after the injection, DECT has evaluated the concentration of NPs in each mice organ. Figure 2 represents the pathway of preparation for in vivo imaging.

### 2.6 Quantification of Au content within tissues

Three hours after the injection of non‐targeted‐AuNPs and targeted‐AuNPs in the mice and DECT scan imaging, the animals were killed. Afterwards, their tissues such as tumour, right and left kidneys, lungs, spleen, heart and liver were extracted and then weighed. Tissues were cut into 2‐mm slices and put in aquilegia solution (2% nitric acid) for 4 h. The amount of realistic Au in various organs was calculated using ICP‐MS [18].

### 2.7 Statistical analysis

One‐way ANOVA was applied for all groups to assess the significance level of the data. *P* ≤ 0.05 was selected as the significant statistical level.

## 3 RESULTS AND DISCUSSION

### 3.1 Synthesis and characterization of NPs

**FIGURE 3:**
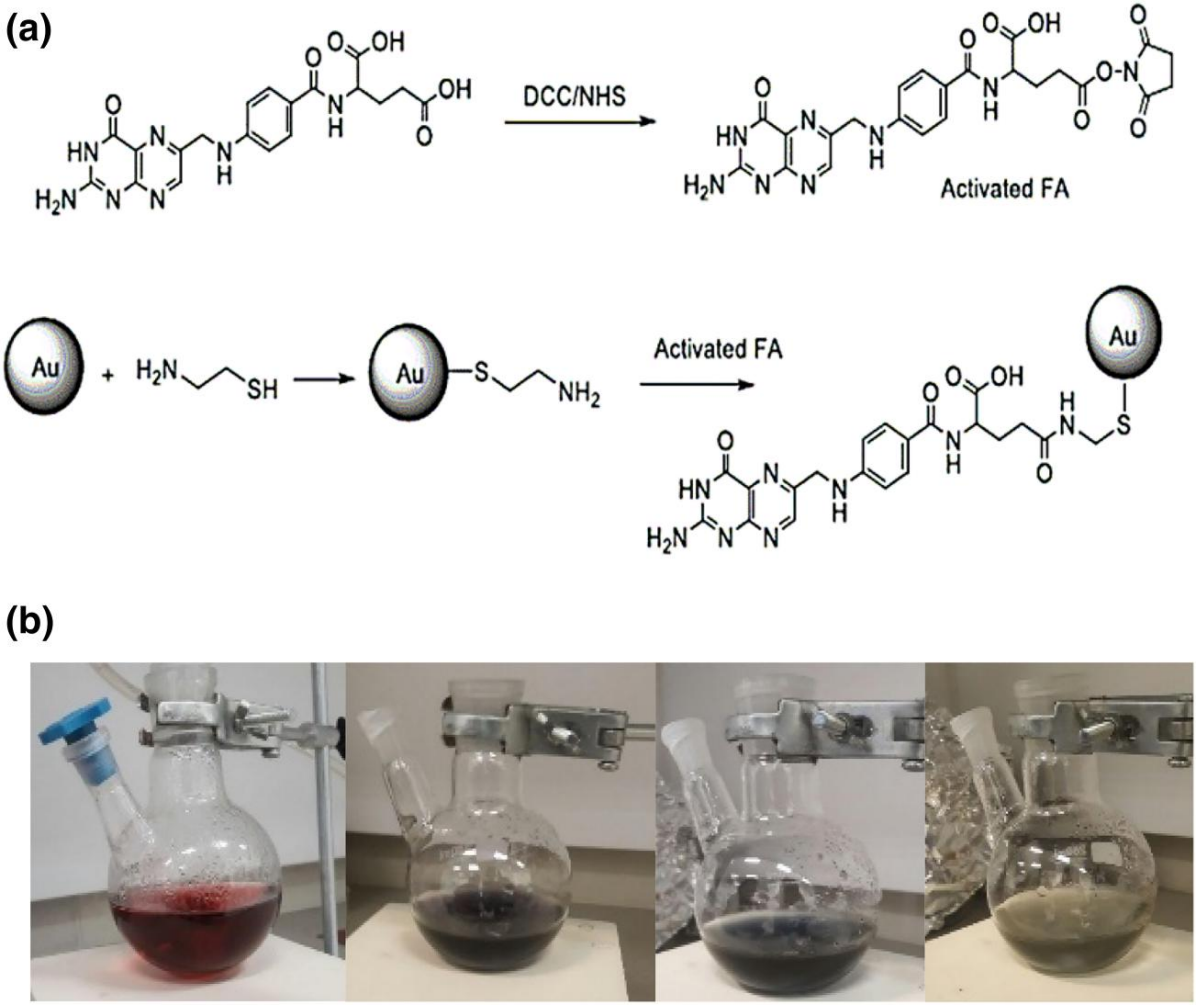
(a) The synthesis of FA‐Cys‐AuNPs using chemical formula and (b) color change of AuNPs after adding the folic acid (left to right)

AuNPs and FA‐Cys‐AuNPs were synthesized, characterized and reported in the previous study [15]. In this research, the results of the injection of NPs in tumoural mice are investigated to show the concentration of Au in different organs using DECT imaging. Figure 3(a) represents the chemical formula of AuNPs to attach activated folic acid with DCC and NHS. Figure 3(b) shows the change of colour AuNPs after adding folic acid and cysteamine. As can be shown, by adding these materials the colour of AuNPs was modified from red to greyish‐blue colour. In the previous study, in brief, suspensions were synthesized and characterized by UV–Vis spectrophotometer to show the surface plasmon absorption at 520 and 705 nm to confirm NPs. The size and morphology of NPs were confirmed by TEM [19]. The mean diameter of AuNPs and FA‐Cys‐AuNPs was 13 and 15 nm, respectively. FTIR spectra confirmed the conjugation of AuNPs to folic acid through cysteamine. The band at 1315 cm^−1^ relies on asymmetric stretching vibration of –NH2 in FA and at 1,677 and 1,627 cm^−1^ based on C–O stretching in carboxyl acids (CA). The bands of 3000 and 3500 cm^−1^ are because of the O–H stretching and NH AVS of FA and Cys, respectively.

**FIGURE 4:**
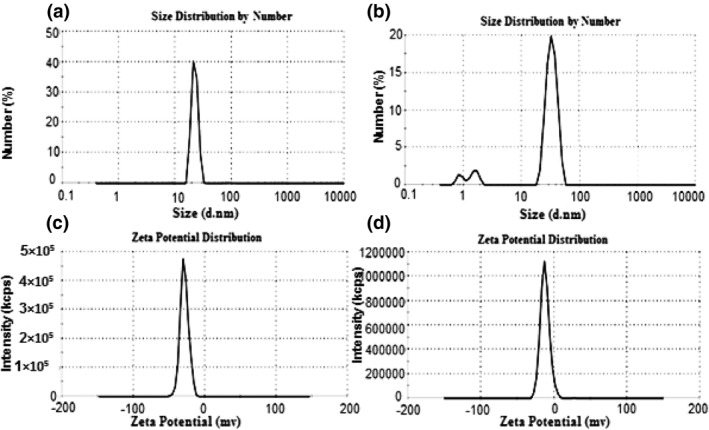
Size distribution of AuNPs (a), and FA‐Cys‐AuNPs (b), zeta potential distribution of AuNPs (c) and FA‐Cys‐AuNPs

As shown in Figure 4(a) and (b), the hydrodynamic size of NPs was confirmed using DLS. The size of AuNPs and FA‐Cys‐AuNPs is 22.4 and 33.8, respectively, that confirmed the results of TEM. Zeta potential was performed to measure the surface charge of NPs that plays an important role in their physiological and aqueous colloidal stability as well as in functionalization. In Figure 4(c) and (d), the zeta potential of AuNPs and FA‐Cys‐AuNPs is (28.5) and (12.5), respectively, and is revealed a good dispersion of NPs.

### 3.2 Haemocompatibility assay

Understanding the cytotoxicity of NPs is necessary to use NP contrast media in vivo and finally apply these materials to the clinic.

Toxicity of targeted‐AuNPs was measured by hematology parameters of blood nude mice. Table 1 shows the toxicity of FA‐Cys‐AuNPs on blood cell parameters. As represented in Table 1, the targeted‐AuNPs probes do not modify the count of blood cells after 14 days. Our findings demonstrated that the developed FA‐Cys‐AuNPs probes are haemocompatible at the Au concentration of 3 × 10^3^ µg/ml.

**TABLE 1:** Blood cell parameters after injection of FA‐Cys‐AuNPs at the concentration of 3 × 10^3^ µg/ml

Parameter	Mice with normal saline (control)	Mice with FA‐Cys‐AuNP	Normal limits	Unit
WBC	7.4	7.8 Normal	4–12	10^3^/µl
RBC	5.3	6.2 Normal	4–6.2	10^6^/µl
LYM	2.5	2.9 Normal	1–5	10^3^/µl
HGB	13	11.1 Normal	11–17	g/dl

**FIGURE 5:**
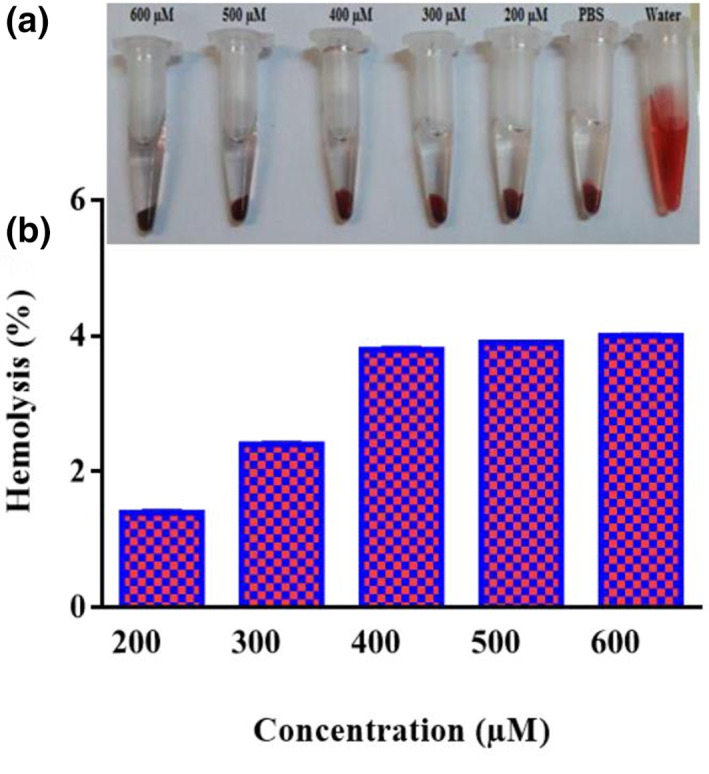
The vials contain blood cells and FA‐Cys‐AuNPs after incubation time for haemolysis assay (a). Haemolysis assay of the haemocompatability of FA‐Cys‐AuNPs at different concentration ranges (200–600 µM) (b)

The haemocompatibility of the nanoprobe was again confirmed by the haemolysis assay. Fresh whole blood was incubated with different Au concentration ranges (200, 300, 400, 500 and 600 µM). As shown in Figure 5, the haemocompatibility of the nanoprobe was the same as the negative control vial (PBS). The blood cells count measurement results corroborate with the above haemolysis assay data.

**FIGURE 6:**
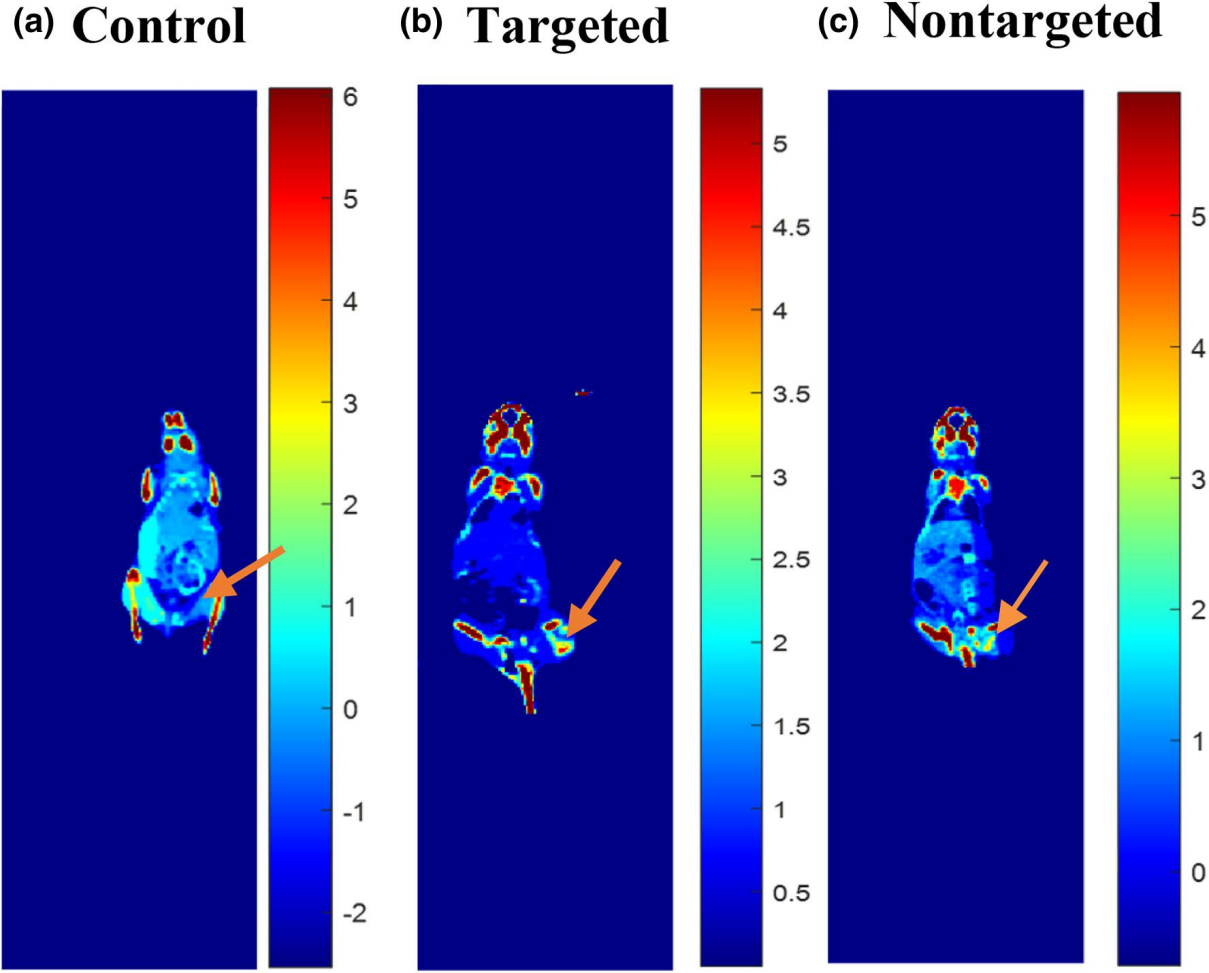
DECT images of (a) control nude mice without injection of NPs, (b) targeted‐AuNPs, (c) non‐targeted AuNPs after 3 h of injection of 3 × 10^3^ µg/ml NPs (orange arrows demonstrated KB tumour)

### 3.3 Targeted DECT imaging of a KB xenografted tumour model in vivo

To study concentration variations of non‐targeted‐AuNPs and targeted‐AuNPs in the head and neck tumoural nude mice model, IV injection through the tail vein was performed. Three hours after injection of NPs (3 × 10^3^ μg/ml, 200 μL), CT imaging was done at two different energy levels (80 and 140 kVp). DECT imaging was performed relying on the sensitivity coefficients (SC) of PMMA phantom calibration. As can be shown in Figure 6 the concentrations of non‐targeted‐AuNPs and targeted‐AuNPs in the various organs were obtained from DECT imaging. Visually the concentration of targeted nanoparticles in the tumour due to the folic acid ligand is more than non‐targeted AuNPs and control. In the previous study, the XAI of targeted NPs compared with non‐targeted NPs was approximately 2 times [20]. Because nasopharyngeal cancer cells have many folic acid receptors on their surface, NPs can enter the cells using endocytosis and lead to elevated concentrations and XAI [14]. Other DECT works have mostly evaluated the separation of two contrast media that simultaneously injected [7, 21, 22, 23]. In the current research, the concentrations of targeted‐AuNPs and non‐targeted‐AuNPs in tumour and other organs of animals were investigated using DECT and then compared with the results obtained from ICP‐MS.

### 3.4 Quantification of Au content within tissues

**FIGURE 7:**
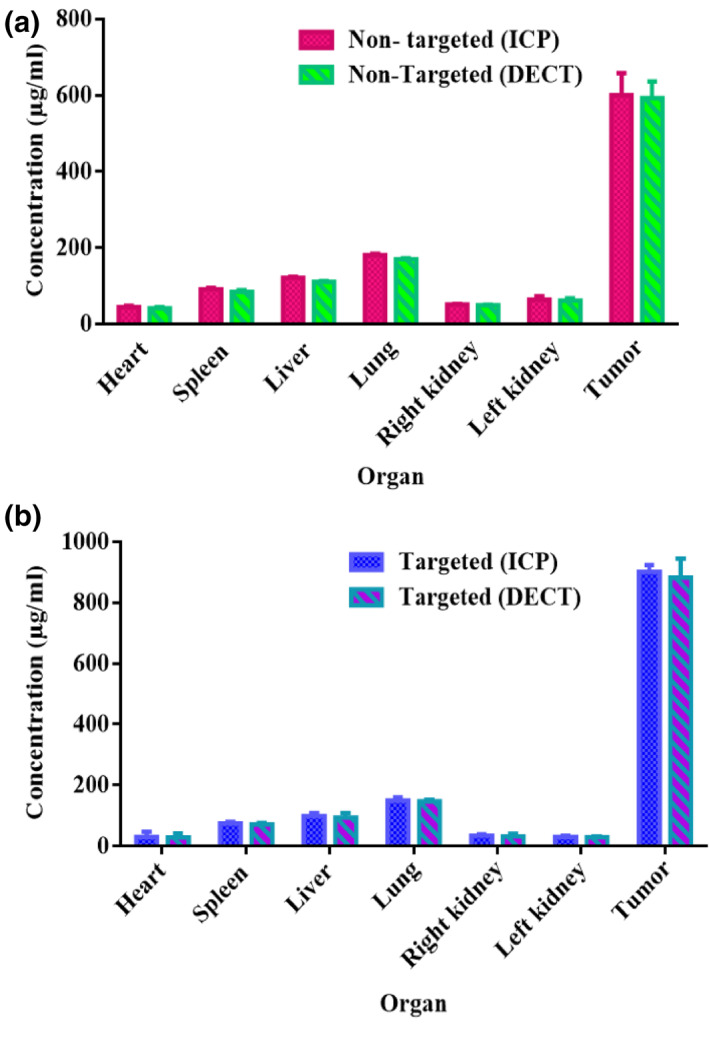
DECT concentration and ICP‐MS analysis of AuNPs (a) and FA‐Cys‐AuNPs (b) after 3 h IV injection in different organs of nude mice

Figure 7(a) and (b) shows the concentration of targeted and non‐targeted AuNPs in several organs and the results of ICP‐MS analysis were compared with DECT imaging. As shown in Figure 7, it was a good result that ICP analysis and DECT imaging nearly to show the similar concentration that confirmed the used DECT algorithm. The highest levels of concentration are seen for both NPs in tumour cells compared to the control group. The concentration of targeted‐NPs was seen more than the non‐targeted cells post‐3 h injection. It is necessary to note that these results were prepared by a hospital CT scan, which has a low resolution (0.625 mm) in contrast to micro‐CT (45 μM) [15]; Since these findings have not been obtained with the micro‐CT, therefore that can be used in the clinic.

## 4 CONCLUSION

The authos applied FA‐Cys‐AuNPs and AuNPs for in vivo DECT images. The haemocompatibility assay was used to measure the blood cell parameters and haemolysis of RBCs of targeted‐NPs at different concentrations. It was suggested that targeted‐NPs had a good haemocompatibility. DECT images were performed at two different energy levels to indicate the concentration of targeted and non‐targeted AuNPs in several organs of the animal. After post‐processing, ICP‐MS analysis confirmed DECT imaging technique that revealed the correct concentration of NPs. Therefore DECT could reveal the distribution of NPs in various organs based on their concentration. At the same concentration, DECT imaging demonstrated, the concentration of targeted‐AuNPs in the nasopharyngeal tumour was higher than non‐targeted‐AuNPs. DECT can decompose the NPs in the body where two contrast agents are injected at the same time.

## CONFLICT OF INTEREST

None declared.
